# New land tenure fences are still cropping up in the Greater Mara

**DOI:** 10.1038/s41598-022-15132-7

**Published:** 2022-07-06

**Authors:** Mette Løvschal, Maria Juul Nørmark, Jens-Christian Svenning, Jake Wall

**Affiliations:** 1 Department of Archaeology and Heritage Studies, Aarhus University, Aarhus, Denmark; 2 Moesgaard Museum, Højbjerg, Denmark; 3 Section for Ecoinformatics and Biodiversity, Aarhus University, Aarhus, Denmark; 4 Center for Biodiversity Dynamics in a Changing World, Aarhus University, Aarhus, Denmark; 5 Mara Elephant Project, Narok, Kenya

**Keywords:** Environmental impact, Sustainability, Animal migration, Ecosystem ecology, Grassland ecology

## Abstract

Expanding and intensifying anthropogenic land use is one of the greatest drivers of changes of biodiversity loss and political inequality worldwide. In the Greater Mara, Kenya, a trend of private land enclosure is currently happening, led by smallholders wishing to protect and uphold their land titles. Here we expand on previous work by Løvschal et al. quantifying the rapid, large-scale development of fencing infrastructure that began in 1985 but has increased by 170% from 2010 onwards. We provide fine-scale analysis of the spatial and temporal trends in fencing using high-resolution Sentinel-2 imagery. The formally unprotected regions have distinctly more fences than the rest of the Mara, one experiencing a 740% increase in fenced land in four years. Conservancies have an effect in stemming fencing but fences crop up within and along conservancy boundaries. We estimate the actual geographical coverage of the fences in the Mara to be 130,277 ha (19% of the total region) using an error margin of 8%, derived by calibrating our satellite mapping with ground-truth data. The study suggests the need for revising community-based eco-conservation efforts and pursuing a richer understanding of the socio-political and historical dynamics underlying this phenomenon.

## Introduction

There is a distressing acceleration in fencing and land fractioning taking place across the globe. The proliferation of fencing in areas that were previously vast, open, largely unfenced grazing land has happened recently across large areas of Inner Mongolia^1^ and historically across Australia and the American Great Plains^2,3^, as well as in northern Europe^4^, with long-term consequences for more-than-human interactions, and reduced wildlife and livestock movement^5^. A comparable situation of large-scale landscape gridding has recently occurred in the Greater Mara, Kenya, East Africa, see in particular the works of Grandin^6^, Løvschal et al. ^7^, Mwangi^8^, Ogutu et al.^9,10^, Said et al.^11^, and Lamprey & Reid^12^. These fences are disrupting physical processes on the ground, but also represent the material expression of underlying sociopolitical, cultural and historical tensions and drivers^13^.

**Figure 1:**
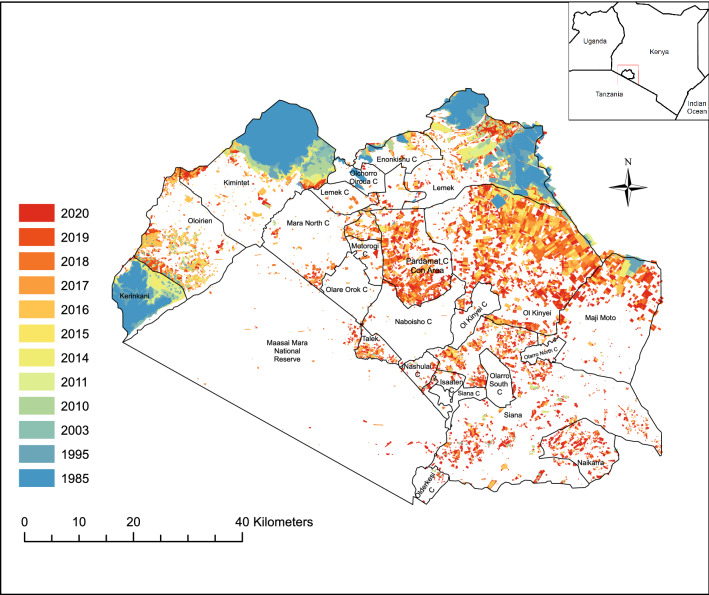
Fences in the Greater Mara, Kenya (1985–2020). Based on a systematic digitization of the fences from Landsat and Sentinel from 1985 to 2016^7^ and 2017–2020 using ArcMap (vs. 10.6.1). Fences are indicated on a sliding color scale described by the legend.

The Greater Mara comprises an area of 668,500 ha situated in the northernmost section of the Serengeti-Mara ecosystem, some 200 km southwest of Nairobi. The Greater Mara consists of 21 administrative regions (Fig. 1), which can be grouped into four types of protected land: The Maasai Mara National Reserve, 16 conservancies (15 community conservancies, one private conservancy-Mara Triangle), one Conservation Area (Pardamat), and remaining formally unprotected land that has no formalized conservation regulatory structure (Fig. 1). A conservation area is a mixed-use area of wildlife and human residency but without the majority of the land parcels receiving lease payments.

The community-based conservation (CBC)^14,15^ institutions in the area work intensively on keeping the different regions free of fences and tearing down already raised fences, to promote wildlife protection. These actions are based on lease contracts with private landowners who receive a regular income for not fencing the land, as well as grazing agreements between pastoralists and conservancies^16^. Conservancies are thereby largely collaborative and community-driven initiatives with a goal of sustainable tourism, although recent studies question their actual community integration^14,15^ and compatibility with pastoral livelihoods^16^. Understanding CBC collective action dynamics associated with eco-conservation, and their underlying paradoxes^13^, is necessary in order to address the fencing issue and why solutions to fragmentation remain unclear. A series of additional drivers appear particularly prominent in incentivizing private landowners to raise and maintain fences, including land-use changes and the need for securing land for landowners, such as including creating grass banks^8,17–19^, and the coincidence with areas of sedentary lifestyle and high population densities.

A previous study of fencing published in *Scientific Reports* in 2017 presented digitized fences from a series of multispectral satellite images (1985–2016)^7^. Satellite-based mapping is often the most viable method over large geographical extents, in impassable areas, or indeed sometimes the only possible method. The study showed a clear acceleration in the speed and scale of fencing in the Greater Mara over the last 30 + years, but also that this increase was a rapid and fairly recent phenomenon. They identified an acceleration in 2010 in the number of fences at key locations, as well as an additional geographical spread in 2014. The study used a conservative methodology that documented an increase in the speed of the spread of fencing, but did not measure areal enclosure of fences, or fence type, although it is estimated most fences appearing across the area are primarily built by small-holders using rectangular plots of c. 0.2–400 ha, and which are typically built from barbed wire with wooden or metal posts. Sometimes simple botanical fences such as cactuses are used. The full spatial extent and density of fencing infrastructure is still poorly understood and the sheer speed and extent at which fences are being built in the Mara make the phenomenon difficult to document as the data quickly become stale.

Løvschal et al.^7^ predicted that accelerating fence construction could have consequences for social and cultural forms of living, as well as for wildlife population size, movement-based ecological processes and species diversity. In combination, the unpredictable, fast-expanding and multicausal nature of the fencing phenomenon makes it difficult to develop regional policies and strategies to balance to human and environmental considerations. Currently, one of the most effective political tools for slowing down the increase in fence development is to support community conservancies, which currently cover c. 114,855 ha in the Mara according to Maasai Mara Wildlife Conservancies Association^20^.

In this article, we seek to update and validate the 2017 study in order to more accurately estimate the fenced areal coverage, spatial expansion patterns and inertia. We provide analysis of the geographic context for fencing as related to land governance, administrative boundaries and historic fencing itself. We base our update of the fencing data on Sentinel-2 images instead of Landsat, improving the spatial resolution from 30 to 10 m (Appendix: Methods). Moreover, we want to validate and qualify data from Løvschal et al.^7^ to provide actual estimations of the geographical patterns of expansion and coverage of enclosed areas. We provide a case study of Pardamat where ground validation data is used to quantify accuracy of the satellite-based approach.

The purpose of this paper is not to argue *for* or *against* fencing, but to provide a spatially informed methodological basis for addressing these issues and negotiating future land-use policies in this area. We acknowledge that an unintended consequence of carrying out such a mapping may contribute to an further increase in the claiming and fencing of the area and its associated disputes and human-conservation-wildlife conflicts. At the same time, accurate data and a data-informed overview of this phenomenon's actual geographical and temporal dynamics are needed for informed policy and decision-making that take environmental and broader socio-political considerations into account.

## Results

The following section assesses our main results in terms of the growth in fenced areas over time relative to 1) types of protection, 2) administrative boundaries, and 3) other fences.

### Fencing relative to land governance

**Figure 2:**
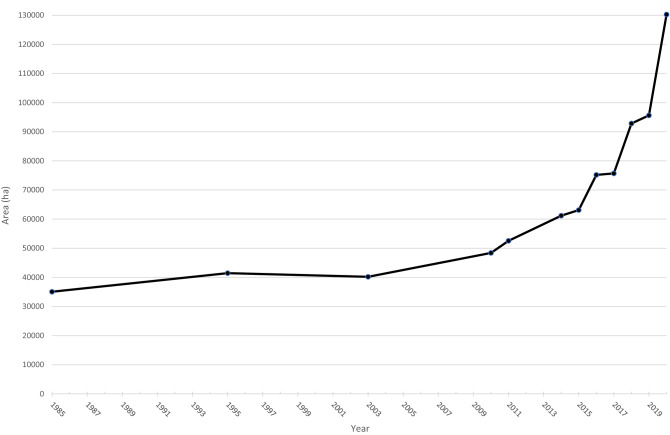
Conservative estimate of the fenced area of the entire Greater Mara, Kenya (1985–2020) expressed in hectares.

Across the Greater Mara, a general growth in fenced areas can be observed throughout the 00 s but in particular over the last decade (Fig. 1). Based on satellite images, 35,067 ha were fenced in 1985, corresponding to c. 5%. In the following 25 years there was only an insignificant increase in fenced plots. However, from 2010, the number of fences suddenly grew rapidly, and in the following period (2015–2020) the fenced area increased even more radically, in an exponential manner (Fig. 2). For example, in 2015 there was 63,112 ha of fenced land; in 2016 this number rose to c. 75,176 ha, corresponding to a c. 20% annual increase. From 2010 to 2020, the ha fenced area increased by 170%. This corresponds to a roughly four times increase in the area enclosed by fences during the study period (1985–2020).

In almost all regions, the number of fences continued to increase in 2019–20 (Fig. 2). The result is a total of 130,277 ha of fenced land in 2020, corresponding to 19% of the Greater Mara.

Hence, there appears to be a building momentum in the expansion of fences in the Greater Mara: those regions that had many fences in 2016 (> 1,000 ha) continue to experience an increase in the area enclosed by fences, with fences spreading almost everywhere in 2020 in particular. Those regions with the fewest fences in 2016 (< 1,000 ha) continue to have relatively few fences.

**Figure 3:**
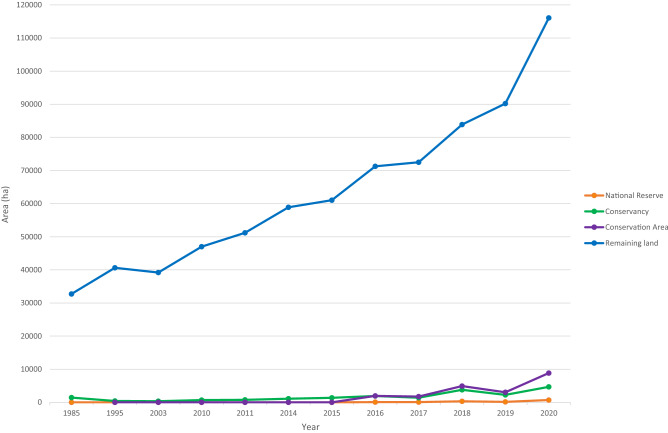
Conservative estimate of the fenced area of the Greater Mara, categorized in terms of level of formal protection (hectares over time from 1985 to 2020).

All types of administrative regions show an increase in fenced land during the study period 1985–2020, with an acceleration in 2010 and a radical expansion since 2014 (Fig. 3).

In the National Reserve hardly any fences were erected from 1985 to 2020 (less than 0.1% fenced land).

**Figure 4:**
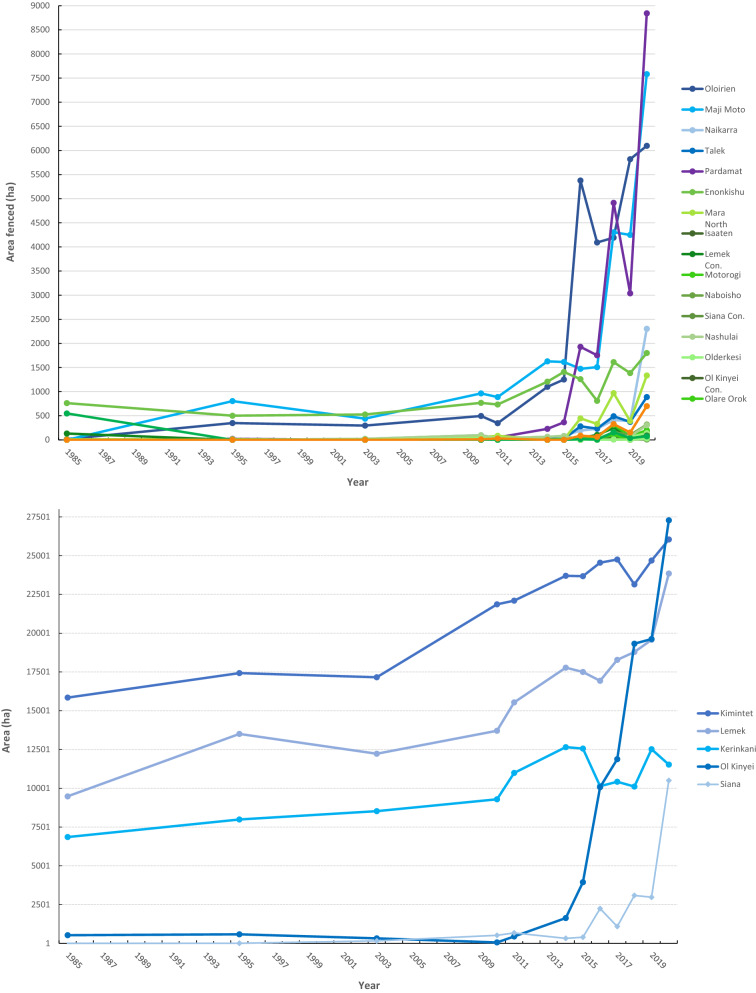
Upper: The areal enclosure of fences in all regions of the Greater Mara in hectares (hectares over time from 1985 to 2020). To illustrate the differences in areal enclosure between the four types of protected land, regions of the type have been assigned the same color ranges. Orange: National Reserve; purple: Conservation Area; green: Conservancy; blue: Remaining land. Lower: The five most fenced regions (cf. formally unprotected land) are illustrated to make it easier to read graph (upper).

In the conservancies, the fenced areas have been relatively constant, with a small increase from 1% coverage in 1985 to 4% in 2020. Most conservancies had only few fences during the entire study period (1985–2020), including Issaten, Nashulai, Ol Kinyei, Naboisho and Olderkesi. In three conservancies (Siana, Olarro North and Olarro South), no fences were registered until 2018, when fewer than 15 ha fences were detected. Even so, these regions with the least fences in 1985–2010 have experienced an increase after 2010 and in particular after 2014 (Fig. 4A).

In the conservation area (Pardamat), changes in the ratio of fenced areas have been more noticeable within the last ten years. It had hardly any fences at all until 2014; then fencing began to increase in an exponential manner from 1,929 ha fenced land in 2016 to 8,842 ha fenced land in 2020, corresponding to an increase of 460% in just four years.

The remaining (formally unprotected) regions in the Greater Mara experienced the most drastic rise in the number of fences in 2010–2020, and the ratio of fenced areas increased from 9% in 1985 to 31% in 2020. The four most fenced regions are discussed below.

The three formally unprotected regions Kimintet (50% of the land is fenced), Kerinkani (77% of the land is fenced) and Lemek (49% of the land is fenced) have distinctly more fenced land than the rest of the Mara throughout the entire study period (Fig. 4B). A fourth region, Ol Kinyei, had hardly any fenced area in 1985–2014, but subsequently fenced land increased exponentially from 3,678 ha in 2015 to 27,283 ha in 2020, corresponding to an 740% increase in fenced land in four years (Fig. 4B). A similar situation was observed for Siana, where less than 1000 ha were fenced in 1985–2015 (< 1%). However, from 2016 to 2020, the amount of fenced land increased from 3 to 12% (Fig. 4B). There are several major urban areas situated in the surrounding unprotected regions (e.g., Sekanani, Nkoilale, Ng’Osuani, Endoinyo Narasha, Aitong, Talek, Lemek) which may explain the historical longevity of the fenced areas here.

Following from the above, there is a clear dependence between the ratio of fences and the formal land governance of a given region. Figure 4A,B shows a remarkable difference in the numbers of fences located within the national reserve, conservancies, conservation areas and remaining land, with the nascent conservancy (Pardamat) and remaining land experiencing the most radical increase since 2010.

### Fencing relative to administrative boundaries

**Figure 5:**
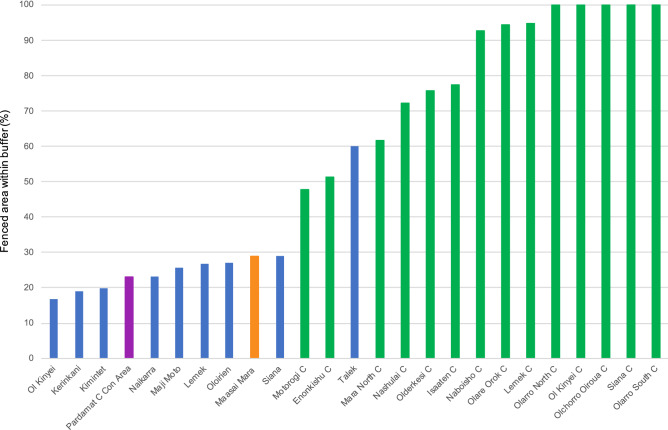
Percentage of fenced area within one kilometer of every border in 2020 relative to the total fenced area of each region of the Greater Mara. Green bars are conservancy areas, the orange bar is the national reserve, the purple bar is the conservation area, and the blue bars are remaining (formally unprotected) regions. The size of the regions is not taken into account in this figure.

Within the conservancies, fences are primarily located near the administrative borders in a buffer zone between conservancies, or between conservancies and remaining land. In 10 out of 16 conservancies, more than 70% of the fences were located within one kilometer of their administrative boundary. In four conservancies, 48–62% of the fences are situated within this buffer zone. In all non-conservancy regions except in Talek, fewer than 30% of the fences were situated within the zone, suggesting a more scattered, ad hoc distribution (Fig. 5).

Outside conservancies, the fences reflected a more ad hoc distribution. More than 70% of the fences were scattered across the area in the sense of each fence not necessarily bordering another fence. An exception is Talek, where 60% of the fences were situated within the one-kilometer buffer zone, a possible explanation being that Talek is a small region (3,920 ha), which increases the likelihood of the fences being situated near its boundaries.

### Fencing relative to other fences

**Figure 6:**
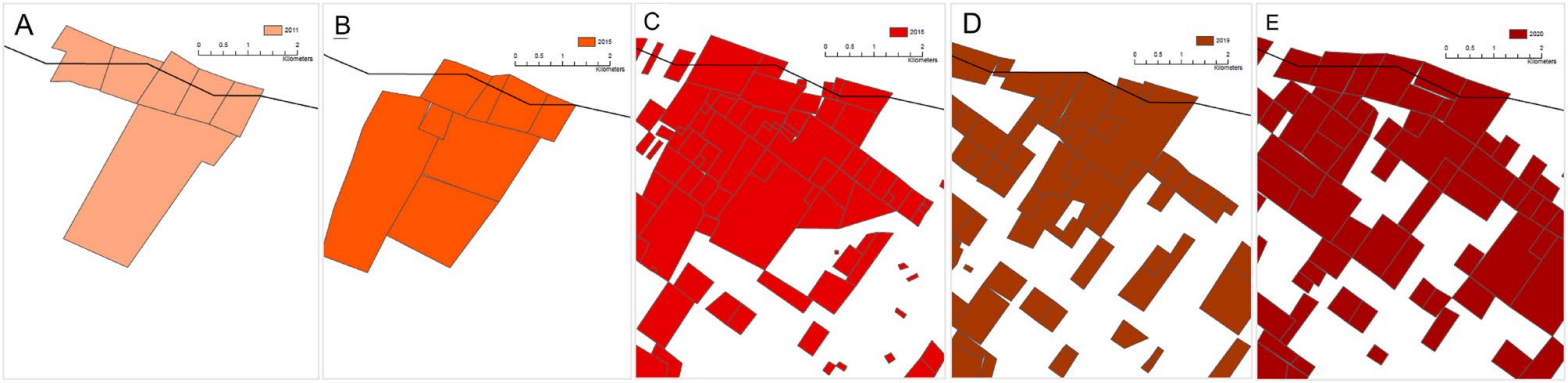
The expansion of fencing around a plot of land in Maji Moto, the Greater Mara, Kenya. (**A**) 2011, (**B**) 2015, (**C**) 2018, (**D**) 2019, and (**E**) 2020. Each section is 5.5 × 5.5 km on the ground. Based on ArcMap (vs. 10.6.1).

**Figure 7:**
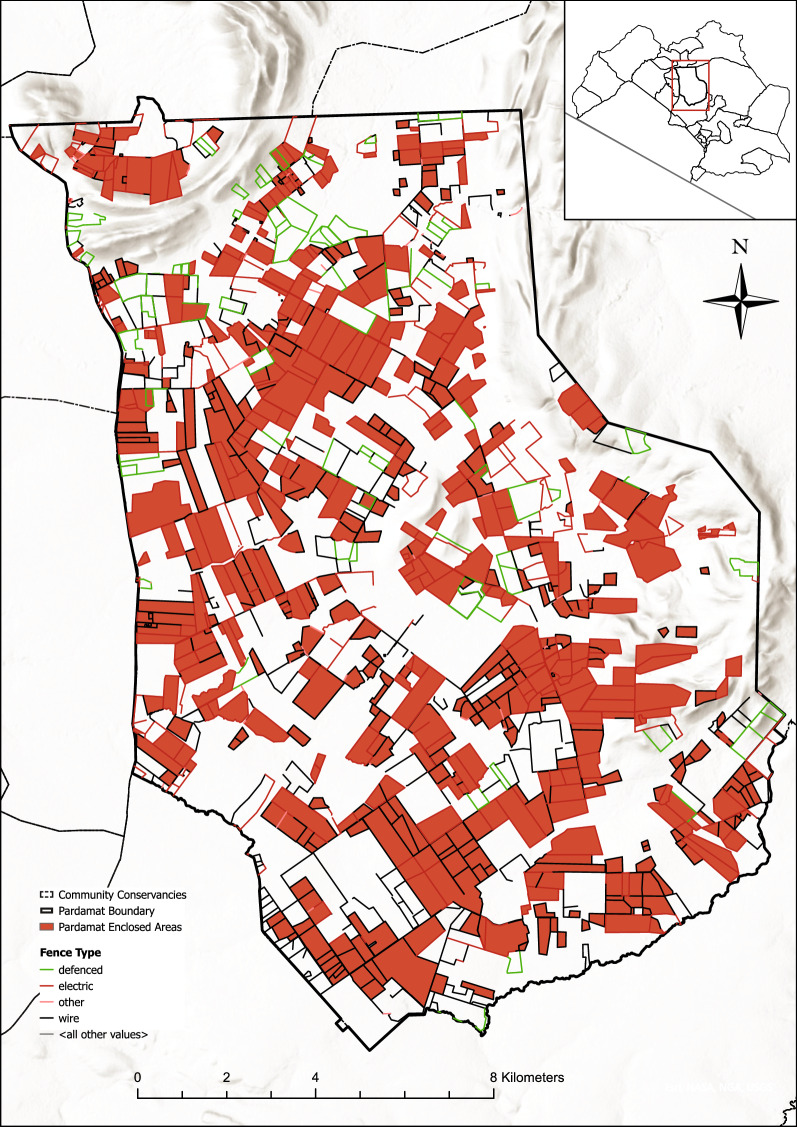
Pardamat fences collected between late September 2019 and September 2021 using GPS ground-truthing. Enclosed areas were calculated by converting recorded fencelines to polygons within Esri ArcGIS Pro vs. 2.9 software^21^ allowing for smaller than 2 m gaps. Green lines represent fences that were removed by landowners during the reported period.

The new fences cropping up in the Greater Mara appear to grow primarily as extensions or add-ons to existing plot fences. In Maji Moto, for example, nearly all fences were repeatedly expanded for each new mapping session between 1995 and 2020 (Fig. 6) suggesting a much more dynamic or fluid picture than what can be seen from Fig. 1, and that is likely relevant to people, livestock and wildlife on the ground. Prior to 2010, the existing plots were divided into increasingly smaller plots, with the size of the overall fenced area staying the same. In the example below (Fig. 6), several smaller fences appeared around the former plot. In 2018 the plot was subdivided into smaller enclosures, and additional plots were added on to it. And from 2019 to 2020, there were so many fences that it questions whether areas that are nearly or entirely surrounded by fenced areas can even be considered unfenced (see also Fig. 7).

In other regions, fences are beginning to fill in open land *between* enclosed plots situated some distance apart. For example, there are now two rather distinctive fronts of fenced plots on the satellite images: a southern-bound front of c. 40 km stretching across Siana and into the National Park, and a northern-bound front starting west of Talek, running through Nashulai, Siana and Maji Moto for at least 52 km.

Some fences have persisted since 2010, others since as long ago as 1995. For example, south of Olorien just north of the border to Kerinkani, a 32-ha enclosed area was surrounded by a few smaller fences in 2010. In the subsequent decade (2011–2019), this fence expanded its area cover to 75 ha. Another example can be found northwest of Enonkishu Conservancy, where the enclosure of a large meat farm, Mara Beef, can be detected on the maps from 2003. In the Maji Moto region, a fence can be observed as long ago as 1995. Across the following 25 years, this fence changed its size and shape, but the entire area remained enclosed during the study period (1995–2020). These examples suggest that the ongoing efforts to erect, expand and maintain fences have now taken place for more than a generation. And that once a fence is erected, it can be difficult to get rid of it again.

### Case study: Pardamat conservation area

Pardamat was formed in 2016 as a mixed-use livestock and wildlife conservation area designed to promote compatibility between traditional husbandry and wildlife-based tourism. Pardamat is comprised of 850 landowners and covers an area of 23,400 ha. Within this region fences spanned 920.6 km in distance including electric (491.6 km (53.4%)), wire (420.6 (45.7%)) or other (8.4 km (0.9%)) with an average fence density of 3.93 km / km^2^ (Fig. 7). Enclosed fence areas covered 8129 ha (34.7%) of the overall region. When compared to the satellite derived method (8840 ha) we find a relatively small, 8% difference in the areal estimates. During the ground truthing period, 96.4 km of fencing were removed by the conservation area management and are not included in the reported values and are responsible for part of the 8% discrepancy between ground-based and satellite-based images (i.e., the satellite digitization occurred before 60 km of fence were defenced).

## Discussion

Following the results presented above, the questions remain: What would it take to break this trend? And how is it even possible to mitigate or reverse the processes of land privatization and colonization that initiated these processes in the first place and their ongoing ramifications?

As mentioned in the introduction, the rapidly expanding processes of land enclosure in Kenya are part of a wider trend occurring not only in East Africa but across the globe^5,22^. In some areas, fences are erected to protect humans, wildlife and livestock; in others, they create massive problems preventing wildlife and livestock from reaching vital water, grazing, and salt resources.

In the Greater Mara, fencing has rapidly increased across vital migration routes as well as into conservancies whose borders are grazed by cattle and sheep/goats^23^, with dramatic effects on wildlife populations and the entire ecosystem. Several studies from the ecological and conservation realm have emphasized the alarming relationship between the increase in fencing and the radical decline of wildlife populations^24^. Fences are being built in areas that are also utilized by the world’s largest and most species-diverse mammal migration—the Mara-Serengeti migration—as well as the somewhat smaller Northern Mara-Loita migration^25^. The Mara-Serengeti migration (1.6 M wildebeest) starts in southern Tanzania and reaches into the Maasai Mara National Reserve, while the Loita migration (100–250 K wildebeest) traverses Ol Kinyei, Naboisho and the Olare Motorogi conservancies and reaches the Loita plains. The pace of the development of new fences across the region is a serious threat to connectivity and movement by wildlife.

Therefore, there is currently a strong need to keep vital areas for migrations and access to water points free from fences. With a view to securing the Greater Mara’s unique ecosystem and its wildlife movements obstructed, we suggest that policy makers take three priorities into account: (1) securing large interconnected habitats by preventing the formation of ecosystem islands; (2) ensuring that large wildlife can move freely within and between the Serengeti and the Loita Plains, including the regions that are currently not formally protected such as Pardamat; and (3) securing both wildlife and livestock access to water and other central resources distributed across the Greater Mara.

At the same time, however, the accelerating spread of fencing is not exclusively an issue of biodiversity. It is also deeply entangled in a series of issues related to histories of land use, policy making and governance, human-wildlife conflicts, eco-tourism, population growth, agricultural expansion, foreign capital and investment, encroachment, and pressure on space. Seen in isolation, the fencing in the Greater Mara may seem like an unprecedented phenomenon which has expanded radically within a decade. But over the long-term perspective, it may be driven in part as a wave of reactions against land histories of marginalization and political decisions since the onset of the colonial era. And the roots of the current boom in fencing can be traced even further back in time than this study permits, including the British colonization (1885–1963)^13^. Paradoxically, although large-scale land allotment has been historically associated with post-colonialist agendas of unequal land distribution and marginalization, the same background is currently incentivizing local landowners to put up fences to secure private rights to land^13^. Hence, in the Greater Mara, by contrast with other regions in East Africa, the propagation of fences across land that was formerly held as communal land is driven strongly by local smallholders.

Collaborative and community-based initiatives to freehold land are very effective in these regions^14^, and are largely a success story. Our study clearly shows the significant effects of the conservancies in halting fencing within these areas, suggesting that protectionist instituions are vital. Expansion of fencing is also not a one-way-street in the sense that fences can and are removed by private landowners themselves with collaborative efforts and pro-conservation incentivizing^26^. At the same time, the eco-tourism industry has potential with a series of ecological downsides, including off-road driving, overcrowding, and garbage disposal issues^27^. Recent evaluations of CBCs in East Africa show rather mixed local community involvement and outcomes stemming from a lack of understanding of embedded political histories related to rights and access, wealth and resource allotment, and top-down policy frameworks^14,15,28^. Hence, long-term solutions creating sustainable livelihoods for both humans and nonhuman animals; including the designation of lands to *live in* and lands to *live from*, are needed in order to ensure a more resilient future human-wildlife coexistence.

Hence, both the ecological, historical, political and socio-economic dimensions of the fencing issue are critical to consider when exploring more resilient forms of more-than-human coexistence and holistic solutions regarding the use of land.

The edge clustering and the scattered distribution (‘*Fencing relative to administrative boundaries’*), can be highly problematic in terms of ensuring human, wildlife and livestock mobility. The many scattered fences increase the risk of wildlife being trapped in barbed wire, for instance. Concentrating plot fences near the administrative borders ensures free movement within the conservancies, and can in this sense be seen as a compromise between the interests of private landowners and conservancies to ensure local land access and areas for people to live and settle. However, such border fences run the risk of turning the conservancies into fenced islands, hindering movement *between* administrative regions. Together with increased grazing pressure in these peripheral areas^23^, this is highly problematic in relation to ensuring pastoral mobility and the freedom of wildlife to roam, as well as influencing wildlife migration routes, in particular the small-scale Northern Mara-Loita migration^24^.

## Methods

**Figure 8:**
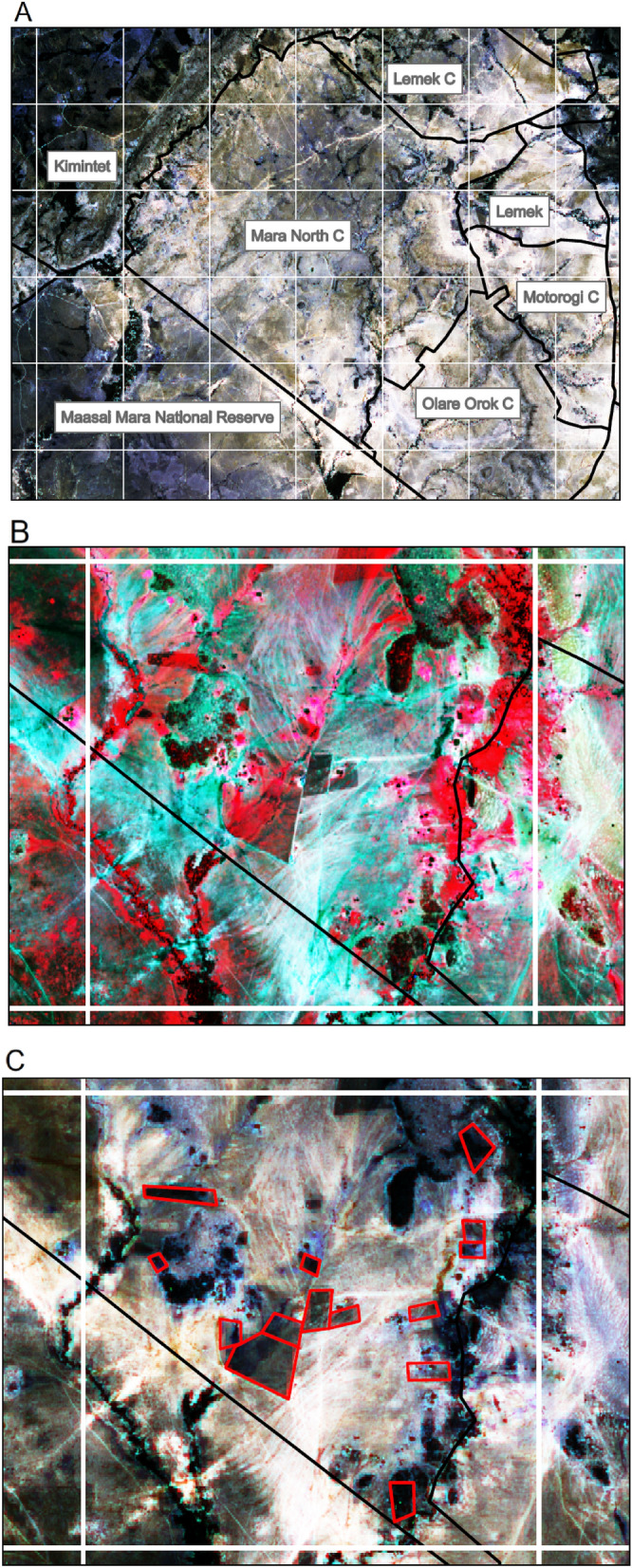
Method illustrated. White lines are the 4.5 × 4.5 km grid; black lines are the regional borders within the Greater Mara; red lines are digitalized fences based on a Sentinel-2 satellite image. (**A**) Mara North seen on Sentinel-2, with normal colors (bands: 4, 3, and 2). (**B**) One grid cell within Mara North (bold white square on Fig. 4A) based on near infrared, red and green, which makes fenced plots stand out as bright red. (**C**) The digitized fences. Based on ArcMap (vs. 10.6.1).

Our analysis is based on two major datasets and their associated set of methods: (1) a satellite-based digitization of fences, following the methods described in Løvschal et al.^7^ (Fig. 8) and (2) spatial vector-data collected by mapping fences on the ground during the period September 2019–September 2021.

In terms of methods (1), first, we selected a satellite image representative of each year in focus and downloaded it via the QGIS plugin from Copernicus Open Access Hub^29^. The digitization of fences during the study period is based on satellite images. The digitalized from 1985 to 2015 were based on images from the Landsat program. Digitization from 2016 to 2020 was based on the Sentinel-2 program. The Sentinel-2 images were all derived from a multispectral imager sensor with a spatial resolution of 10 m, greatly improving the Landsat 30-m resolution.

Although studies have found that data from the Landsat program and Sentinel-2 program best correlates in spring/summer, images for this study were extracted for February or March^30^ This was solely based on the comparability with the previous data^7^. An allowed cloud cover range was specified to be < 5%, and most images had cloud cover of < 2%.

Second, each image was made into three copies. Each copy went through a separate processing changing the band-colors of the images to enhance differences in vegetation. (1) Red, green and blue (natural colors): Band 1 was set to show band 4, band 2 was set to show band 3, band 3 was set to show band 2. (2) Red and green (near infrared): Band 1 was set to band 8, band 2 was set to band 4, band 3 was kept at band 3. (3) False color composite image with the Principal Component Tool in ArgGIS. Each copy was set to “*Histogram Equalize*” in the stretch type settings and the statistics setting was set to “*From Current Display Extent*”. Everything else was kept as it was. In Løvschal et al.^7^, two more images were used for digitization: a normalized difference vegetation index (NDVI) layer, and a short-wave infrared (SWIR) layer. These two layers were not used in this paper (data for 2016–2020) because our previous experience revealed that they did not improve the mapping of fencing significantly.

Third, we overlaid the map with a cell grid of 4.5 × 4.5 km to ensure the same precision throughout the digitizing of fences (Fig. 8A). Each map was analyzed one grid cell at the time, enabling systematic progress. The image was zoomed in so only one grid cell was visible at the time. This posed a risk of overlooking fences situated on or across these four visible grid lines. The advantages of this method, however, exceed this risk, since it enables a systematic assessment at the same zoom level and of the total area, grid by grid. In order to assess if a structure on the map was a fence or not, the three processed copies of the same image were consulted. First the normal image was analyzed and scanned for squared structures, which would be darker than the surrounding vegetation (Fig. 8B). Next, the near infrared image was used as a confirmation or rejection of this structure as being human-made (a fence). High amounts of vegetation would be red and dark red. – And in areas of intensive grazing or agriculture, there would be less or no or even colored vegetation, indicating a fenced area. Lastly, the *principal component* image was turned on to further confirm or reject whether it was a fence. A square was drawn around the structure and when all structures within the focal grid cell was digitalized, we moved on to the next grid cell (Fig. 8C). This method ensured that the total areas was analyzed and only one at a time. This method was conducted by one person for the 2016–2020 data, and other person for the 1985–2016 data. To ensure compatibility between these two datasets, a series of learning images were used prior to the analyses. Independently, both persons digitalized fences for 2016, with Landsat data and Sentinel-2 data, resulting in a > 5% difference. Even though the above-described method is still subjective, it is currently the quickest and easiest method available to digitalize fences from satellite images.

Fences were mapped using Esri ArcGIS (ArcMap vs. 10.6) software by Esri^31^.

Regions of the Greater Mara are either categorized as a conservancy (green), national reserve (orange), conservation area (purple) or remaining (formally unprotected) area (blue), as illustrated by Fig. 3. Currently, both Lemek and Siana represent a mix of conservation areas and formally unprotected land, so both regions are present twice in Fig. 4, using “Con.” as an abbreviation for “conservancy”.

Conservancy boundaries have changed slightly since 2016^7^. Oloisukut is now officially a MMWCA conservancy. Such minor changes in the exact position of the administrative boundaries across the last few decades mean that some fences that were situated inside conservancies 10 years ago are now situated right outside them. However, to provide comparability between the studies, we have kept the former boundaries (14 instead of 16 established conservancies) for replicability and to be able show the changes in time. Moreover, since we document vegetation shifts, not the actual line of the metal wires, there can sometimes be significant delays between when fences are erected, and we can detect consequent land-use changes. However, our comparison of the satellite and ground-based methods shows that our satellite-imagery-based method accurately estimates fencing extent. Semi or fully automated machine-learning techniques may, in the future, enable faster and more extensive mapping capacity. Moreover, there are various opportunities for producing better-quality data and accessing larger quantities of openly available data, such as the recently published Landscape Dynamics database (Supporting Information).

In terms of methods (2), between September 2019 and September 2021 Mara Elephant Project (MEP) collected ground-based fence-line information as part of ongoing ground-based human-elephant conflict mitigation efforts. Each fenceline was physically visited on the ground and mapped using the ‘TerraChart’ Android mobile GPS application^32^. The TerraChart mobile application collects linear fence features as ArcGIS polylines and were classified as Wire’, ‘Electric’, or ‘Other’. A 2 km^2^ overlay grid was used to guide the fence collection and to ensure complete coverage across the landscape. Once all fences were mapped within a grid, the grid was marked complete with the date of completion. Data are stored and curated in the ‘Landscape Dynamics’ database (Supporting Information) and available for download through the Esri ArcGIS Online platform^33^.

We performed an analysis of the extent and density of fences in the Pardamat conservation area to compare with the aforementioned satellite digitization technique by first projecting data into the WGS84 UTM Zone 36S projection and clipping to the Pardamat boundary extent. Esri ArcGIS Pro (vs. 2.9 software^21^ was then used to calculate enclosed areas from the polylines using a 2 m tolerance (i.e., gaps 2 m or smaller in the fence were considered to be closed). Specifics of the analysis can be found in the *Pardamat_Fencing.ipynb* Jupyter Notebook available in the Supporting Information. A readme file details setup of a notebook environment to reproduce this analysis.

## Conclusion

In this article, we have shown that fencing keeps spreading in the Greater Mara at an alarming pace, resulting in an acceleration and the initiation of a trajectory of physical enclosure in which boundaries breed more boundaries. Our study has also shown the ways in which fencing spreads in relation to (1) forms of landholding, (2) administrative boundaries, and (3) other fences. The analyses suggest that although there has been a broad-scale and continuing rise in the number of fences, the most explosive rise has taken place in (formally) unprotected regions such as Pardamat, Ol Kineyi and Maji Moto in particular. Within conservancies, fences tend to cluster along the administrative boundaries. Outside the conservancies, fencing tends to crop up in a rather rapid, chaotic fashion.

Our study shows that fencing keeps spreading despite a series of community-based and regional political initiatives and eco-conservation efforts and information– suggesting that such initiatives are ineffectual or even potentially co-driving this phenomenon. On the ground, the incentives to create fences are not only increased by the visible, physical pressure of fences cropping up around many smallholders; but by an equally perceived pressure from unfenced areas as well as from conservancies and international interests. In this situation, increased information on ecosystem and wildlife protection as well as CBC interventions falls short in restraining this phenomenon^14,34^. Instead, it calls for other ways of thinking and developing (community-based) conservation policies and initiatives and involvement by external actors, not only in the Greater Mara but concerning eco-conservation on a much broader scale. We have concentrated here on the spatial and temporal dynamics of fencing; however, our study highly suggests the need for a much richer and complex understanding of the socio-political and historical dynamics underlying this phenomenon.

## Data and access

The data presented in this study is available on request from the corresponding author. The fencing data is partly available (1985–2016) from the Landscape Dynamics database (Supporting Information). All satellite images were collected via the QGIS plugin from Copernicus Open Access Hub (QGIS vs. 10.3.2009). The images used in this study were all derived from a multispectral imager sensor from the Sentinel-2 satellite with a spatial resolution of 10 m. All images were downloaded during spring 2019, as digitization work progressed, except one image from 2017, which was downloaded in April 2020 because of access problems. This is negligible in the analyses.

## References

[1] Taylor JL (2006). Negotiating the grassland: The policy of pasture enclosures and contested resource use in inner Mongolia. Human Org..

[2] Netz, R. *Barbed Wire: An Ecology of Modernity* (1st ed.). (Wesleyan University Press, 2009).

[3] Greer A (2012). Commons and enclosure in the colonization of north America. Am. Hist. Rev..

[4] Løvschal M (2020). The logics of enclosure: deep-time trajectories in the spread of land tenure boundaries in late prehistoric northern Europe. J. Roy. Anthropol. Inst..

[5] Tucker MA (2018). Moving in the Anthropocene: Global reductions in terrestrial mammalian movements. Science.

[6] Grandin, B. E. The Maasai: socio-historical context and group ranches in *Maasai Herding*: *An Analysis of the Livestock Production System of Maasai Pastoralists in Eastern Kajiado District, Kenya* (eds Bekure, S., de Leeuw, P. N., Grandin, B. E. & Neate, P. J. H.) 21–39 (ILCA, 1991).

[7] Løvschal M (2017). Fencing bodes a rapid collapse of the unique Greater Mara ecosystem. Sci. Rep..

[8] Mwangi E (2007). The Puzzle of Group Ranch Subdivision in Kenya’s Maasailand. Dev. Chang..

[9] Ogutu JO, Piepho HP, Dublin HT, Bhola N, Reid RS (2009). Dynamics of Mara-Serengeti ungulates in relation to land use changes. J. Zool..

[10] Ogutu JO, Owen-Smith N, Piepho HP, Said MY (2011). Continuing wildlife population declines and range contraction in the Mara region of Kenya during 1977–2009. J. Zool..

[11] Said MY (2016). Effects of extreme land fragmentation on wildlife and livestock population abundance and distribution. J. Nat. Conserv..

[12] Lamprey RH, Reid RS (2004). Expansion of human settlement in Kenya’s Maasai Mara: what future for pastoralism and wildlife?. J. Biogeogr..

[13] Løvschal M, Gravesen ML (2021). De-/fencing grasslands: Ongoing boundary making and unmaking in postcolonial Kenya. LAND.

[14] Galvin KA, Beeton TA, Luizza MW (2018). African community-based conservation: a systematic review of social and ecological outcomes. Ecol. Soc..

[15] Salerno J (2021). Adaptation and evolution of institutions and governance in community-based conservation. Conserv. Sci. Pract..

[16] Bedelian C, Ogutu JO (2017). Trade-offs for climate-resilient pastoral livelihoods in wildlife conservancies in the Mara ecosystem, Kenya. Pastoralism.

[17] Homewood K (2001). Long-term changes in Serengeti-Mara wildebeest and land cover: Pastoralism, population, or policies?. Proc. Natl. Acad. Sci..

[18] Li W (2020). Accelerating savanna degradation threatens the Maasai Mara socio-ecological system. Glob. Environ. Chang..

[19] Nyariki DM, Mwang’ombeb AW, Thompson DM (2009). Land-use change and livestock production challenges in an integrated system: the masai-mara ecosystem, Kenya. J Hum Ecol.

[20] https://maraconservancies.org/

[21] Available at: https://pro.arcgis.com/en/pro-app/latest/get-started/download-arcgis-pro.htm

[22] McInturff A (2020). Fence ecology: Frameworks for understanding the ecological effects of fences. Bioscience.

[23] Løvschal M, Håkonsson DD, Amoke I (2019). Are goats the new elephants in the room? Changing land-use strategies in Greater Mara Kenya. Land Use Policy.

[24] Ogutu JO (2016). Extreme wildlife declines and concurrent increase in livestock numbers in Kenya: What are the causes?. PLoS ONE.

[25] Veldhuis MP (2019). Cross-boundary human impacts compromise the Serengeti-Mara ecosystem. Science.

[26] Andersson D (2021). Dynamics of collective action to conserve a large common-pool resource. Sci. Rep..

[27] Bhandari MP (2014). Is tourism always beneficial? A case study from Masai Mara national reserve, Narok, Kenya. Pac. J. Sci. Technol..

[28] Nelson F (2021). Progress or regression? Institutional evolutions of community-based conservation in eastern and southern Africa. Conserv. Sci. Prac..

[29] QGIS 10.3, QGIS Development Team, 2019. QGIS Geographic Information System. Open-Source Geospatial Foundation Project. Available at: http://qgis.osgeo.org.

[30] Arekhi M (2019). Comparative evaluation of the spectral and spatial consistency of Sentinel-2 and Landsat-8 OLI data for igneada longos forest. Int. J. Geo-Inf..

[31] Available at: https://www.esri.com/en-us/arcgis/products/arcgis-desktop/resources

[32] Available at: https://play.google.com/store/apps/details?id=org.maraelephantproject.terrachart)

[33] Available at: https://maraelephant.maps.arcgis.com/home/item.html?id=0a57b9fba82f4c86b452ffa758b6fb8e

[34] Alexander SM, Andrachuk M, Armitage D (2016). Navigating governance networks for community-based conservation. Front. Ecol. Environ..

